# Bioinformatics analysis of the wheel treadmill test on motor function recovery after spinal cord injury

**DOI:** 10.1002/ibra.12006

**Published:** 2021-12-09

**Authors:** Qiu‐Lin Wang, Ting‐Ting Li, Chang‐Le Fang, Bao‐Lei Zhang

**Affiliations:** ^1^ School of Anesthesiology Southwest Medical University Luzhou Sichuan China; ^2^ Department of Anesthesiology, Institute of Neurological Disease, West China Hospital Sichuan University Chengdu China; ^3^ Department of Experimental Zoology Kunming Medical University Kunming Yunnan China

**Keywords:** bioinformatics, motor recovery, spinal cord injury, treadmill test

## Abstract

This study aimed to explore the possible target and mechanism of the wheel treadmill (WTM) test for motor function recovery of spinal cord injury (SCI). Rats were divided into sham, control and WTM groups to establish an SCI mode. Rats in the WTM group were trained on the WTM test, and Basso–Beattie–Bresnahan (BBB) scores were determined. The samples were collected, and mRNA sequencing was conducted to determine the changes in gene expression. The coexpressed genes were screened to construct a protein–protein interaction (PPI), followed by the Kyoto Encyclopedia of Genes and Genomes pathway and Gene Ontology function enrichment analysis, and the differentially expressed genes (DEGs) volcano map and hub gene expression heat map were constructed using R language. The BBB scores in the control and WTM groups increased with time, with the WTM group scoring higher than the control group. The results of rat spinal cord tissue sequencing showed that a total of 1679 DEGs were screened in the sham and control groups; 928 DEGs and 731 overlapping genes were screened in the WTM and control groups. The key genes were identified by PPI analysis. One hundred and thirty‐three genes were found to be overlapping by combined analysis of spinal cord sequencing data and BBB scores of rats at Week 7. The top 10 DEGs from high to low were T*yrobp, Rac2, Cd68, C1qb, Aif1, Cd74, Spi1, Fcer1g, RT1‐D*A, and *Ccl4*. The terms with the highest enrichment scores were microglia‐mediated positive regulation of cytotoxicity and major histocompatibility complex class II protein complexes. Treatment with the WTM test promotes recovery of motor function after SCI in rats by modulating intercellular communication and immune function.

## Graphical abstract

The Basso–Beattie–Bresnahan scores in the control and wheel treadmill (WTM) groups increased with time, with the WTM group scoring higher than the control group. The WTM test promoted motor function recovery of spinal cord injury (SCI) rats by regulating the expression of Tyrobp, Rac2, Cd68, Aif1, and other genes. Treatment with the WTM test promotes recovery of motor function after SCI in rats by modulating intercellular communication and immune function.

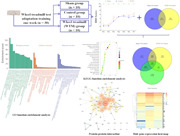

## 1 INTRODUCTION

Spinal cord injury (SCI) is any injury to the spinal cord that can lead to sensory and motor dysfunction. Spinal cord contusion is the most common form of SCI. The site of injury determines the symptoms of SCI, such as pain, urinary incontinence, intestinal dysfunction, motor and sensory dysfunction, muscle dystonia, the appearance of pathological reflexes, and so forth.^1^ According to the World Health Organization, 250,000–500,000 new SCI patients are diagnosed worldwide each year.^2^ There are 300,000 SCI patients, with approximately 17,000 new cases arising every year in America.^3^ SCI is divided into primary injury and progressive secondary cascade injury. The damage caused by the initial physical force causes the primary damage, such as compression, shearing, tearing and so on. The primary injury determines the severity of the injury. Secondary SCI is a delayed and progressive tissue injury following primary SCI. Its main pathological features include ischemia, expression of proapoptotic signals and infiltration of peripheral inflammatory cells. Over time, the release of cytotoxic fragments such as proinflammatory cytokines and deoxyribonucleic acid (DNA) leads to the formation of the injury microenvironment. When the disease progresses to the chronic stage, scar formation of astrocytes around the combined lumens can severely hinder the regeneration and repair of the injury.^4^ Due to the low plasticity of the central nervous system (CNS), neuregeneration ability is limited, and the recovery of neural function after SCI is very difficult. At present, the treatment of SCI is mainly aimed at protecting and promoting nerve regeneration and functional recovery. So far, it is hard to find ways to cure with SCI.^5^

At present, physical exercise is used to enable the recovery of motor function after SCI. Since 1980, when Forssberg et al. first reported that the wheel treadmill (WTM) test significantly improved motor recovery in patients with SCI,^6^ the WTM test has been widely used worldwide as a method to improve functional walking after SCI. Moreover, Battistuzzo et al. pointed out that the WTM test promoted the recovery of motor function more significantly than other sports such as swimming in different animal models of SCI with different types of exercise training.^7^ It is worth noting that patients with SCI have increased rates of glucose intolerance, decreased insulin resistance and diabetes. Chilibeck et al. reviewed relevant studies and found that the WTM test can improve the body's ability to process glucose and thus reduce the risk of developing diabetes.^8^ In addition, some studies have reported that the WTM test can reduce the incidence of cardiovascular disease complications by reducing blood lipid levels in patients with SCI.^9^ Also, Shin et al. investigated differences in gene expression in mice with SCI and found that genes related to neuroplasticity and angiogenesis were highly expressed in mice after the WTM test.^10^ In summary, the recovery of motor function after SCI by the WTM test may be achieved by action on nerve‐ and vascular‐related genes and related signaling pathways, but the specific molecular mechanism by which the WTM test promotes motor function recovery has not been fully clarified.

In this study, by observing the recovery of motor function after the WTM test in rats with SCI, this study aims to analyze the gene expression and explore the possible mechanism of its action, so as to provide a possible new approach for the treatment of motor function in patients with SCI.

## 2 MATERIALS AND METHODS

### 2.1 Experimental animals and grouping

A total of 30 adult female healthy Sprague–Dawley (SD) rats of specific pathogen free (SPF) grade, weighing 250–300 g, were purchased from the Experimental Animal Center of Kunming Medical University. The rats were housed in cages and given sufficient food and water during the feeding period. The ambient temperature was 23 ± 2°C, with a natural light–dark cycle. Thirty SD rats were randomly divided into three groups of 10 rats each, as follows: the sham group (only opening of the spinal cavity to expose the spinal cord, no SCI model was established and no WTM test was conducted), the control group (SCI model setup and wound suture, without the WTM test), and the WTM group (WTM) (SCI model setup and wound suture, with the WTM test). All experimental procedures were approved by the Animal Care & Welfare Committee of Kunming Medical University (kmmu2019005).

### 2.2 Establishment of the SCI model in rats

The SCI model of rats was established using an impactor.^11^, ^12^, ^13^ Thirty SD rats were weighed and recorded, and anesthetized by an intraperitoneal injection of pentobarbital (60 ml/kg).^14^ After a deep coma was induced, hair of the rats was removed above and below the 10th thoracic vertebra for skin preparation. The rats were fixed in the prone position on an ultraclean experimental manipulation platform, and the skin preparation site was wiped and disinfected with iodophor. With the T10th thoracic vertebra as the center of the incision, an incision about 1–1.5 cm long was made along the center of the rat's back, followed by blunt separation of the fascia and muscle. Then, the muscles on both sides were incised with a scalpel centered on the spine to fully expose the spinous processes and laminae of T9‐T11, avoiding damage to the surrounding blood vessels and nerves and leaving the intact dura as the injury area. The rats were then transferred to HATTRAS (pci‐3000) for horizontal and prone fixation. The diameter of the round head of the HATTRAS (pci‐3000) was 2 mm, the speed was 4 m/s, the depth was 1.5 mm, and the dwell time was 0.1 s. First, the position of the impactor was adjusted to the midline of the dura surface (depth = 0), and then the computer‐controlled was used to shock the dural and spinal cord tissues of the rats. Spinal cord hematoma and sudden stiffness of the hind limbs (or loss of movement of both hind limbs) are the criteria for successful injury. After the impact, if there was any bleeding, it was immediately stopped and the muscle layer and skin of the rat were sutured layer by layer; then, the sutured wounds were cleaned with iodophor after suturing. The SCI model was established in both the control group and the WTM group. In the sham group, only the T9‐T11 spinal eminence and part of the lamina were surgically removed to expose the spinal cord; no SCI was induced.

After the SCI modeling surgery was completed, the rats were placed on a thermostatically heated blanket to prevent hypothermia and waited for rats' awakening.^11^, ^12^ After the rats awakened, they were transferred to cages for observation, during which they were fed and watered normally and given certain amounts of sunflower seeds every week to supplement their nutrition. On the second day after surgery, each rat was injected with 80,000 units of sodium penicillin (0.9%) intraperitoneally to control infection, and bladder massage was performed once a day in the morning and evening to aid urination until the rats were able to urinate on their own. The stuffing was changed every 3 days to ensure that the rat cage was dry and hygienic. During this period, if the lower body of the rats was found to be moist and the lower limbs were edematous and ulcerated, it was washed with warm water and wiped with iodophor.

### 2.3 WTM test

All rats were subjected to 20 min/day of WTM test adaptation training 1 week before the establishment of the SCI model. Only rats in the WTM group were subjected to formal training on the second day after the establishment of the SCI model. The rats of the WTM group were placed on the treadmill and the speed was set to 5 rpm/min until the rats could move their hind limbs slowly. During the training period, the recovery of hind limb motor function was gradually accelerated, with the maximum acceleration up to 14 rpm/min. Training was performed once a day for 20 min each time, and lasted for 7 weeks.^15^

### 2.4 Basso–Beattie–Bresnahan (BBB) score

The scoring team consisted of three experienced scorers, and the scoring process was performed using a double‐blind protocol. The rats were placed in a closed environment at a room temperature of 20°C and allowed to crawl freely. The BBB scores were determined once a week at 9:00 a.m. The scorers observed the hind limb activity of the rats carefully and assigned a score for the motor function of the left and right hind limbs of each rat in each group according to the BBB scale. The hind limb motor function score was 0–21, and the higher the score, the better the hind limb motor function; if the hind limb did not move on its own, a score of 0 was assigned.^16^ SPSS 21.0 statistical software was used to conduct statistical analysis of repeated measures of variance (ANOVA) for BBB score data, which were expressed as mean ± standard deviation. The pandas library was used to extract, connect, and export data. Scipy library was used to complete normal distribution judgment and correlation statistical analysis of BBB score data (Pearson correlation analysis was used for data with a normal distribution, and Spearman correlation analysis was used for data with a non‐normal distribution).

### 2.5 Tissue sampling

After completion of the WTM test and recording of BBB scores, the spinal cords of rats in each group were sampled. Pentobarbital and saline were prepared 1 day before sampling, and surgical instruments and operating tables were disinfected. Thirty SD rats were weighed, recorded, and anesthetized by an intraperitoneal injection of pentobarbital (60 ml/kg). After administration of anesthesia, the rats were placed in a supine position and disinfected. The abdomen, diaphragm, and chest were cut open to expose the heart. A fixed needle was perfused from the left ventricle, the aorta was inserted, the right auricle was incised, and the infusion device was connected. The rats were infused with precooled normal saline until the liver became white and the flushing fluid became clear. Then, a bone biter was used to open the medullary cavity of rats. The upper and lower ends of the scar at the SCI were taken, rinsed with saline and placed in 1.5 ml PE tubes and then frozen in a −80°C refrigerator.

### 2.6 Eukaryotic mRNA sequencing

Total ribonucleic acid (RNA) was extracted from scar tissue of the spinal cord. There were three groups in the expriment (WTM, control, sham), and three samples from each group were selected for eukaryotic mRNA sequencing, resulting in a total of nine samples. Nanodrop2000 was used to detect the concentration and purity of extracted RNA, agarose gel electrophoresis was used to detect RNA integrity and Agilent2100 was used to determine the RNA integrity number (RIN). A–t base pairing was conducted between magnetic beads with Oligo (dT) and ploy A, and mRNA was separated from total RNA. The enriched mRNA was added into fragmentation buffer, and appropriate conditions were selected to randomly split the mRNA into small fragments of about 300 bp. Under the action of reverse transcriptase, the fragment mRNA was reversed to synthesize one‐strand cDNA with random primers, and then two‐strand cDNA was synthesized to form a stable double‐strand structure. End‐Repair Mix was added to make the flat End, and then an A base was added to the 3ʹ End to connect the Y‐junction. After connecting the adapter, the products were purified and segmented. Polymerase chain reaction amplification was performed with the segmented products, and the final purification was performed out of the cDNA library.^17^ The Illumina HiSeq sequencing was performed.

### 2.7 Analysis of Illumina HiSeq sequencing results

The raw data of Illumina HiSeq sequencing results were statistically controlled for data quality control, and the raw sequencing data were filtered to obtain high‐quality sequencing data. In RNA sequencing (RNA‐SEQ) analysis, gene expression levels were calculated using RNA‐Seq by Expectation‐Maximization (RSEM) (clean reads counts) that were located in genome regions. Fragments per kilobase of exon model per million mapped fragments (FPKM) were used to measure gene expression levels. After obtaining Read counts of genes, multiple (≥2) samples were analyzed (the sham group vs. the control group; the control group vs. the WTM group), differentially expressed genes (DEGs) between samples were identified, differential expression hypothesis values, false discovery rate (FDR) and log2FC (Sample B/Sample A) values were calculated and regulatory values (Sample A is the control, UP is upregulated and DOWN is downregulated) were obtained according to the changes in gene expression levels.

### 2.8 Analysis of gene expression levels in combination with BBB score data at Week 7

The expression data of all genes sequenced from the spinal cord scar tissue were matched with the corresponding BBB score data at Week 7, and then the two sets of data were examined to find the correlation between gene expression and BBB scores. In python, the pandas library was used for one‐to‐one data extraction, matching, concatenation and data export, and the RE library was used to complete the data name matching in the process of data extraction and concatenation. The Scipy library was used to complete the normal distribution judgment and correlation statistical analysis (Pearson correlation analysis was used for normally distributed data and Spearman correlation analysis was used for non‐normally distributed data), and finally, the statistical analysis results were exported in the form of tables. The condition of data filtering was set as *p* < 0.01 in the exported table, and then the *R*‐value columns (relational series) were sorted from the smallest to the largest by their absolute values.

### 2.9 Screening of differential genes

The sequencing results showed differential gene expression in the control group versus the sham group and the control group versus the WTM group. In the personalized filter, the filter condition was set to FDR < 0.01, |log2FC| > 1.5. Then, all the genetic data in the file that satisfy the filter condition were sorted according to the FDR value from smallest to largest.

### 2.10 Screening of coexisting DEGs

The list of genes screened according to certain conditions was selected for intersection in the Venn 2.1 database (https://bioinfogp.cnb.csic.es/tools/venny/), that is, two genes with different group expression specificities were selected, and then the result of the obtained gene intersection was showed through Venn plot.

### 2.11 Construction of protein–protein interaction (PPI) networks and functional enrichment analysis

To further explore the functional and interaction relationships of DEGs, PPI networks of DEGs were constructed using the String database (https://string-db.org/). The PPI networks of hub genes were mapped using Cytoscape 3.7.2 software. The Metascape database (http://metascape.org/) was used for Gene Ontology (GO) enrichment and Kyoto Encyclopedia of Genes and Genomes (KEGG) pathway enrichment analysis of differentially expressed proteins, and the *p*‐value cutoff was set at 0.05.

### 2.12 Construction of a volcano map and a hub gene expression heat map

The table files of gene expression differential analysis results were imported into R language and the corresponding working paths were set. After the table files were called by the openxlsx package, the scatter color, map size, and upper and lower partition conditions were set and the differential gene volcano maps were drawn using the ggplot2 package. The average expression of different genes in each group is summarized into a table using the same gene pairing. After calling the table files using the openxlsx package, the expression‐level data in the tables were normalized by logarithmic transformation. Then, the gene expressions are plotted by setting the corresponding color fields with the Pheatmap package in R language.

## 3 RESULTS

### 3.1 Comparison of BBB scores between the three groups

**Figure 1:**
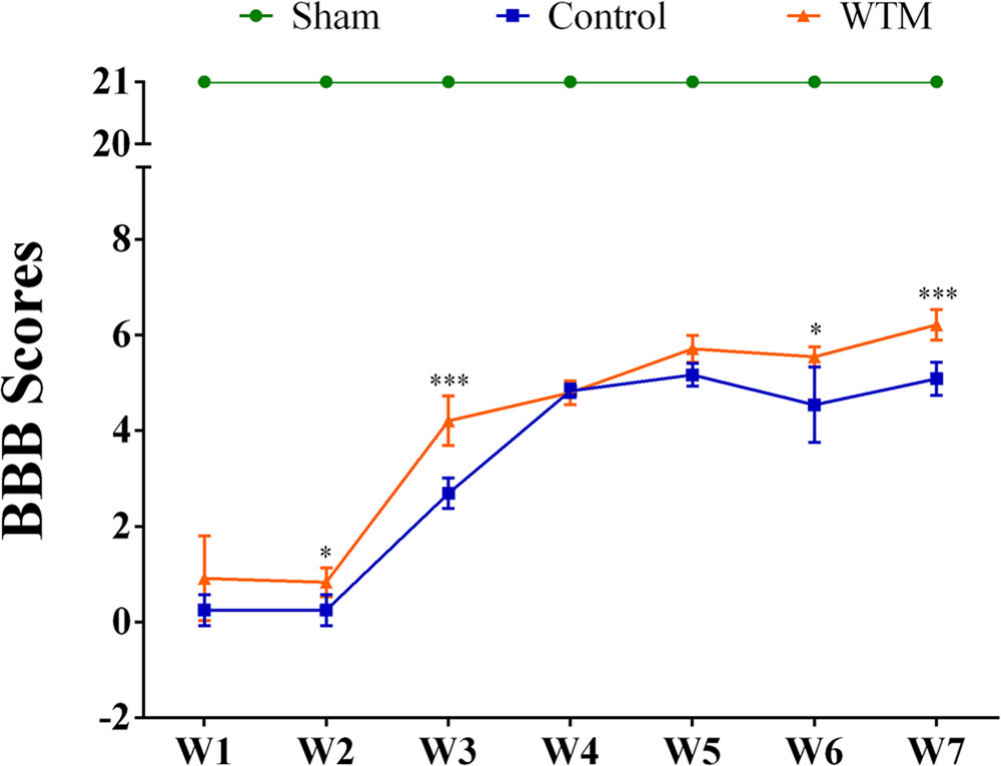
Comparison of BBB scores between the three groups. *p < 0.05, ***p < 0.001, there was a significant difference between the WTM group and the control group. The BBB score of the WTM group was significantly higher than that of the control group. Statistical methods: repeated‐measures ANOVA. The error line extends to the most extreme data point, in the range of 1.5 quartiles. BBB scores, Basso–Beattie–Bresnahan scores; W, week(s); WTM, wheel treadmill [Color figure can be viewed at wileyonlinelibrary.com]

In the sham group, since there was no SCI, the motor function was not impaired; the BBB score of the sham group was maintained at 21 points. The BBB scores of both the control group and the WTM group showed a gradual increase, indicating that the hind limb motor ability of both groups recovered to different degrees with time (Figure 1). The control group was the SCI model group, without the WTM test. The BBB scores of the rats in the control group were significantly lower than those in the sham group, suggesting that the BBB score of the control group was significantly lower than that of the sham group because the SCI model was established and the motor function was impaired. Rats in the WTM group received wheel treadmill test. Compared with the control group, the BBB scores in the WTM group were significantly higher than those in the control group, especially at the second, third, sixth, and seventh weeks, suggesting that the WTM test could promote the recovery of hind limb motor ability SCI rats. Also, there was an interaction between the WTM test and time (Figure 1). With an increase in scorekeeping, the BBB scores of rats all increased to different degrees, and the increase in scores in the WTM group was higher than that in the control group.

### 3.2 Screening results of DEGs

**Figure 2:**
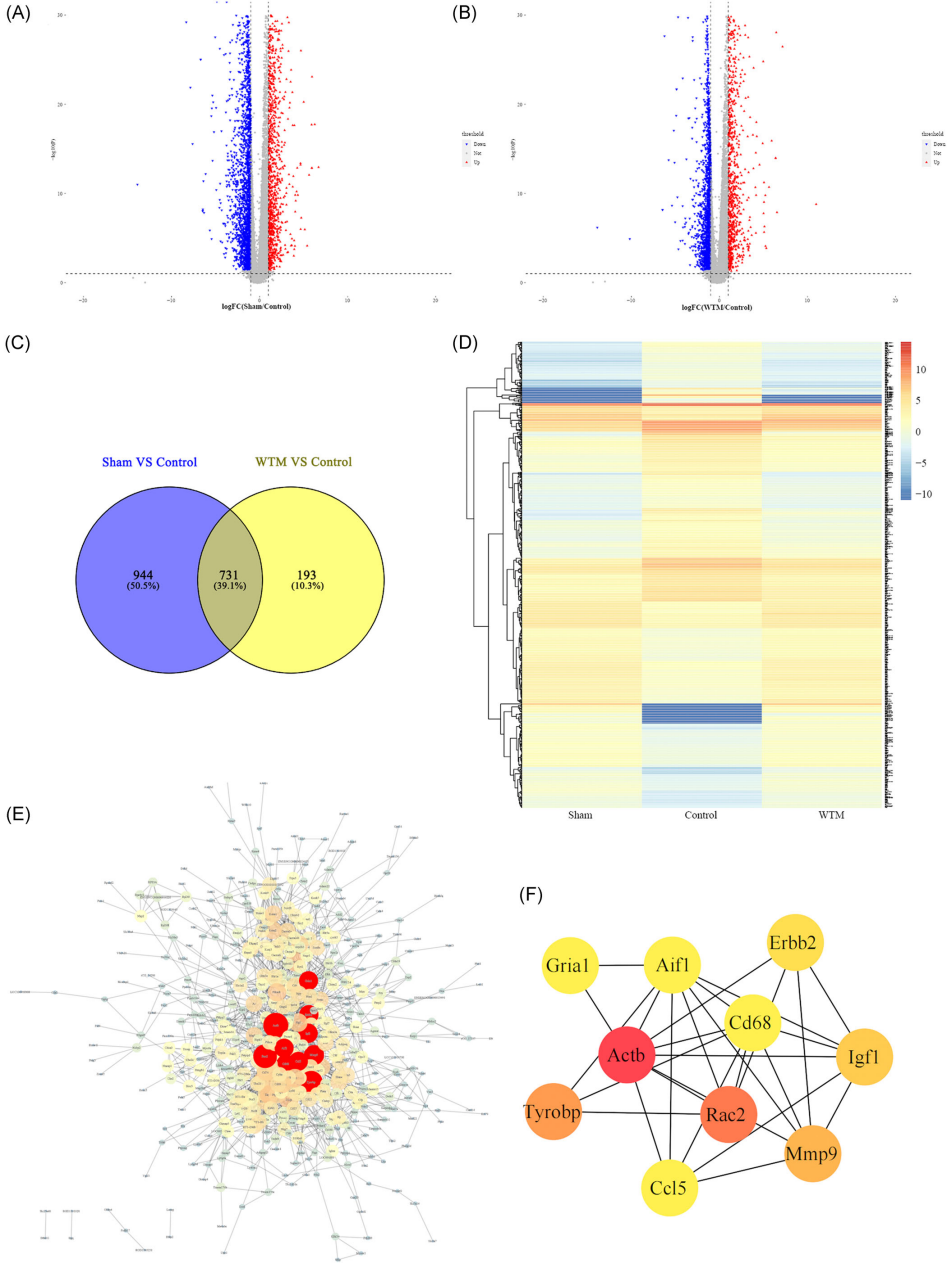
DEGs of SCI rats in each group. (A) and (B) Volcano grams of gene expression (A: in the sham group and the control group; B: in the control group and the WTM group); blue inverted triangles represent downregulated genes, red positive triangles represent upregulated genes and gray dots represent unchanged genes. (C) Venn diagram of DEGs between the sham group versus the control group and the control group versus the WTM group. (D) Heat maps of DEGs expression between the sham and control groups, and the control and WTM groups. (E) Interaction of DEPs between the sham group versus the control group and the control group versus the WTM group. Each circular node represents a protein target, and each connecting line represents the interaction between two target proteins. The larger the node, the higher the core degree of the protein target, and the thicker the connection line, the stronger the interaction between the two proteins. (F) Interaction diagram of the top 10 protein targets in degree. DEGs, differentially expressed genes; SCI, spinal cord injury; WTM, wheel treadmill [Color figure can be viewed at wileyonlinelibrary.com]

By collating and analyzing the sequencing data of rat spinal cord tissues, we determined the screening criteria for significant DEGs as follows: *p* < 0.05, |log2FC| ≥ 1. A total of 3838 differential genes were screened in the sham group and the control group, of which 1188 genes were upregulated and 2650 genes were downregulated (Figure 2A). Between the control group and the WTM group, a total of 2917 differential genes were screened, including 1016 upregulated genes and 1901 downregulated genes (Figure 2B). To perform precise screening of genetic variants, the precise screening criteria of FDR < 0.01 and |log2FC| = 1.5 continued to be followed. A total of 1679 differential genes were screened between the sham and control groups, and 928 differential genes were screened between the WTM and control groups. Subsequently, a total of 731 DEGs were screened by cross‐selection using the Venn database for the above two differential gene groups (Figure 2C). Heat maps showed the changes in gene expression levels of the 731 DEGs in the sham, control, and WTM groups (Figure 2D). Protein interaction analysis and hub gene screening results identified 493 protein interactions. There were 1687 PPI relationships between the genes (Figure 2E). The top 10 genes were *Actb, Rac2, Tyrobp, Mmp9, Igf1, Erbb2, Aif1, Ccl5, Gria1*, and *Cd68* (Figure 2F).

### 3.3 DEG screening with BBB scores

**Figure 3:**
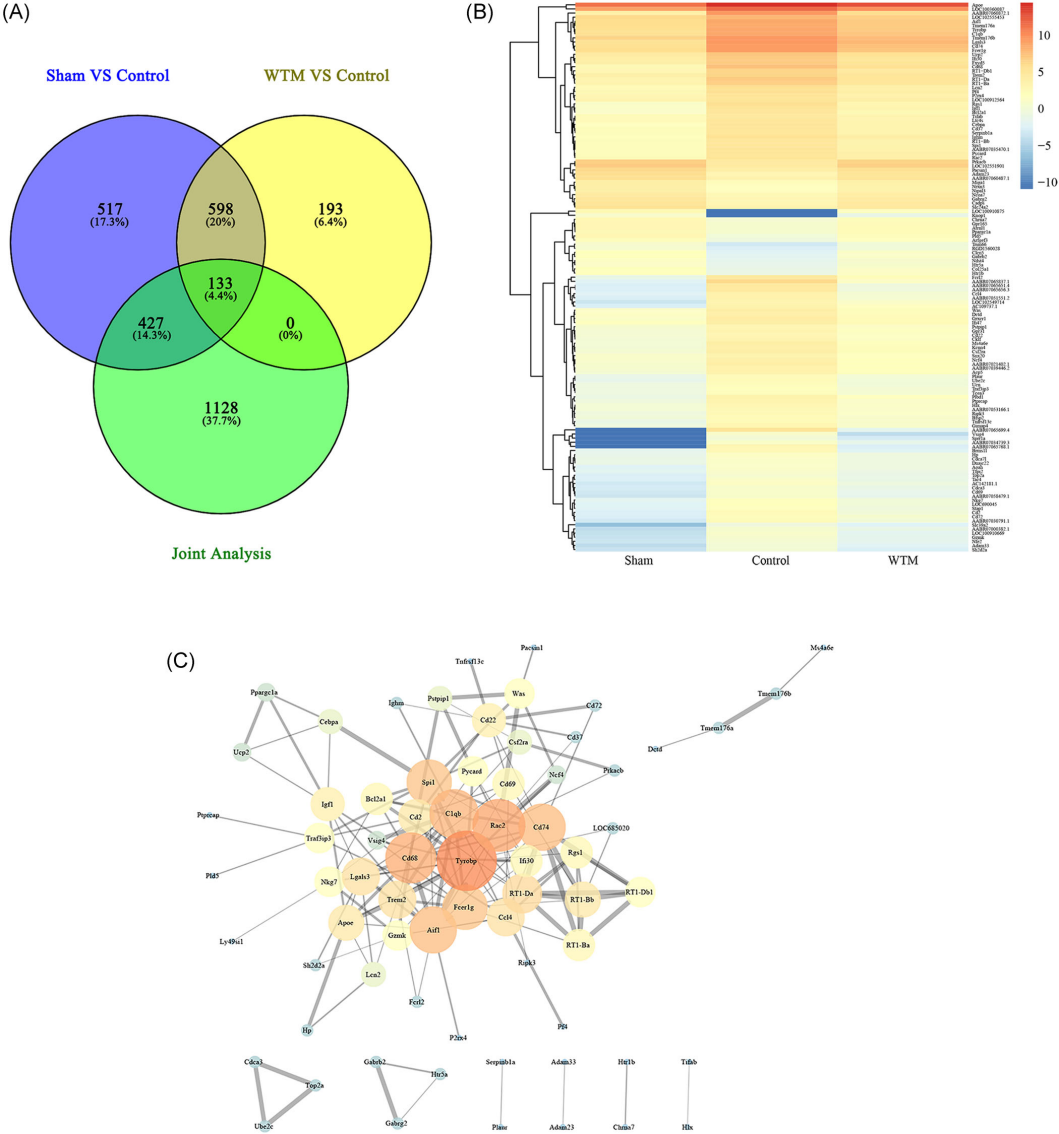
Combined analysis of differentially expressed genes in rats with SCI. (A) Venn diagram of coexisting differentially expressed genes and peak and area correlation analysis. (B) Heat map of expression changes in DEGs in Venn intersection. (C) Gene protein interaction diagram of Venn intersection DEGs. Each circular node represents a protein target, and each connecting line represents the interaction between two target proteins. The larger the node, the higher the core degree of the protein target, and the thicker the connection line, the stronger the interaction between the two proteins. DEG, differentially expressed gene; SCI, spinal cord injury; WTM, wheel treadmill [Color figure can be viewed at wileyonlinelibrary.com]

A total of 7880 DEGs were identified, including 2502 positively related genes and 5378 negatively related genes, after a combined analysis of spinal cord sequencing data and BBB scores of rats at 7 weeks. Then, according to the qualification of the |*R*| ≥ 0.9, 1688 related genes were screend and coexist with the front screened 731 DEGsand protein interactions using Venn database for intersection. A total of 133 genes associated with BBB scores at Week 7 in rats with SCI were selected, that is, the DEGs in both the sham group and the control group and the DEGs in the control group and the WTM group (Figure 3A). Next, we screened 133 DEGs. Gene expression changes were measured in the control group and the WTM group, respectively, and heat maps of gene expression changes were drawn (Figure 3B), in addition to protein interaction analysis and hub gene screening (Figure 3C); 70 interactions of protein genes were found. There were 168 PPI relationships between them. These interacting protein genes were sequenced according to the degree value. The top 10 DEGs from high to low were T*yrobp, Rac2, Cd68, C1qb, Aif1, Cd74, Spi1, Fcer1g, RT1‐D*A, and *Ccl4*.

### 3.4 Function and pathway analyses of DEGs in rats with SCI

**Figure 4:**
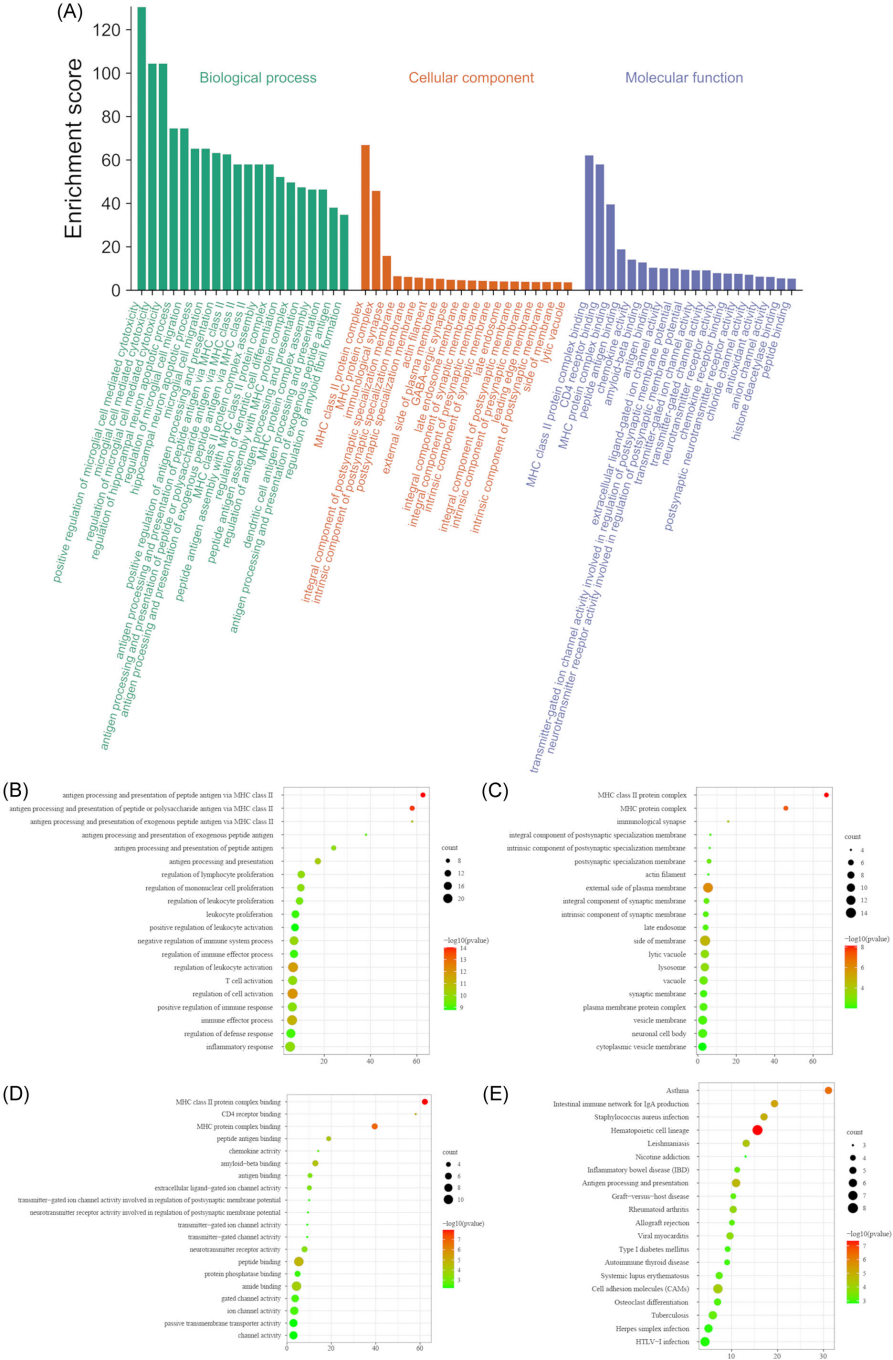
GO enrichment analysis and KEGG pathways. (A) The top 20 BP, CC, and MF of GO enrichment analysis. (B) The top 20 BP of GO enrichment analysis. (C) The top 20 CC of GO enrichment analysis. (D) The top 20 MF of GO enrichment analysis. (E) The top 20 KEGG signaling pathways. The size of dots in the figure represents the number of proteins enriched in this term. BP, biological process; CC, cellular component; GO, Gene Ontology; KEGG, Kyoto Encyclopedia of Genes and Genomes; MF, molecular function [Color figure can be viewed at wileyonlinelibrary.com]

The biological process (BP), cellular component (CC), and molecular function (MF) of GO enrichment analysis were determined using the Metascape database (Figure 4A–D). The top 20 BP mainly involved regulation of microglial cell‐mediated cytotoxicity, the hippocampal neuron apoptotic process, microglial cell migration, antigen procession and presentation (peptide, polysaccharide antigen, and exogenous peptide antigen), amyloid fibril formation, and major histocompatibility complex (MHC) protein complex assembly (mainly class II) (Figure 4A: green part and Figure 4B). The top 20 CC were the MHC class Il protein complex, the MHC protein complex, immunological synapse, the integral component of the postsynaptic specialization membrane, the intrinsic component of the postsynaptic specialization membrane, the postsynaptic specialization membrane, the actin filament, the external side of the plasma membrane, the integral component of the synaptic membrane, the intrinsic component of the synaptic membrane, late endosome, side of the membrane, lytic vacuole, lysosome, vacuole, the synaptic membrane, the plasma membrane protein complex, the vesicle membrane, neuronal cell body, and the cytoplasmic vesicle membrane (Figure 4A: orange part and Figure 4C). The top 20 MF were MHC II class protein complex binding, cluster of differentiation (CD4) receptor binding, MHC protein complex binding, peptide antigen binding, chemokine activity amyloid‐beta binding, antigen binding, extracellular ligand‐gated ion channel activity, channel activity involved in the regulation of postsynaptic membrane potential, receptor activity involved in the regulation of postsynaptic membrane potential, transmitter‐gated ion channel activity, transmitter‐gated channel activity neurotransmitter receptor activity, peptide binding, protein phosphatase binding, amide binding, gated channel activity, ion channel activity, passive transmembrane transporter activity, and channel activity (Figure 4A: purple part and Figure 4D). The top 20 signaling pathways assessed by KEGG pathway analysis were asthma, the intestinal immune network for IgA production, *Staphylococcus aureus* infection, hematopoietic cell lineage, leishmaniasis, nicotine addiction, inflammatory bowel disease, antigen processing and presentation, graft‐versus‐host disease, rheumatoid arthritis, allograft rejection, viral myocarditis, type I diabetes mellitus, autoimmune thyroid disease, systemic lupus erythematosus—cell adhesion molecules (CAMs), osteoclast differentiation, tuberculosis, herpes simplex infection, and HTLV‐I infection (Figure 4E).

## 4 DISCUSSION

After rats were grouped and after SCI modeling, BBB evaluation results for 7 consecutive weeks showed that the WTM test could promote the recovery of hind limb motor function in rats with SCI. The rats were then sacrificed, and the scar tissue of the spinal cord was obtained for eukaryotic mRNA sequencing. Through Illumina HiSeq sequencing, the Venn 2.1 database, the String database, the Metascape database and R language, the results showed that the WTM test may promote the recovery of motor function in rats with SCI by regulating the expression of genes such as *Actb, Rac2, Tyrobp, Mmp9, Igf1, Erbb2, Aif1, Ccl5, Gria1*, and *Cd68*. In addition, the BBB scores indicated that there are interactive effects of the WTM test and time on locomotor function recovery of SCI. In other words, the recovery of spinal cord function in the rats was the result of a combination of time and treadmill training. Therefore, to explore the mechanism of WTM on SCI rats more accurately, 133 DEGs of the intersection between BBB scores and gene sequencing of rats in each group were acquired through the Pathy. Protein interaction analysis of these 133 DEGs showed that the top 10 DEGs from high to low were *Tyrobp, Rac2, Cd68, C1qb, Aif1, Cd74, Spi1, Fcer1g, RT1‐DA*, and *Ccl4*. The terms with the highest concentration of BP, CC, and MF of the 133 DEGs were positive regulation of microglial cell‐mediated cytotoxicity and MHC, particularly the MHC class II protein complex. KEGG results showed that most of the proteins were enriched in hematopoietic cell lines, asthma, and IgA‐producing intestinal protein networks. The results of KEGG and GO suggest that the WTM test may promote the recovery of motor function in rats by regulating the intercellular communication function and immune function.

### 4.1 Comparison of BBB scores among the three groups

Time after SCI has a certain influence on BBB scores.^18^ With time, the BBB scores of both the control group and the WTM group showed a gradual increase, indicating that with time, the motor ability of SCI rats would be enhanced.^19^ Compared with the control group, the weekly BBB score of the sham group was significantly higher than that of the control group, indicating that the hind limb motor ability of the control group was significantly decreased. In addition, the BBB score of the WTM group was also significantly higher than that of the control group, especially at the second, third, sixth, and seventh week, indicating that the rotating WTM test can promote the recovery of hind limb motor ability in SCI rats. These results were consistent with previous studies, which provides a guarantee for us to obtain the correspoding gene sequencing analysis accurately.^8^, ^9^ It is worth noting that from the BBB score results, it can be concluded that there are interactive effects between the two factors of runner training and time. Therefore, BBB scores of consecutive 7 weeks were included for further analysis to identify the key gene targets of the wheeled treadmill test on spinal cord function recovery in the case of time extension.

### 4.2 Screening of DEGs

On combined analysis of spinal cord sequencing data and seventh week BBB scores of rats, the top 10 DEGs in degree values were obtained, including the *Tyrobp, Rac2, Cd68, C1qb, Aif1, Cd74, Spi1, Fcer1g, RT1‐DA*, and *Ccl4*. The expression levels of the above 10 genes were significantly downregulated in the WTM group compared with the control group. *Tyrobp* is the gene that encodes tyrosine kinase binding protein (also known as DNAX‐activating protein of 12 kDa, DAP12),^20^ a transmembrane signaling polypeptide that is a homodimer bound by disulfide bonds, an immune‐related gene.^21^, ^22^, ^23^ In the analysis of key genes of prognostic value in gastric cancer, Jiang indicated that *Tyrobp* is related to positive macrophage activation, tumor necrosis factor (TNF) production regulation and tolerance induction regulation, and may play an immunosuppressive role in CD8 T cells and macrophages.^24^ DAP12 mainly plays a role in signal transduction and inflammation, and can interact with *Rac2* in the PPI network.^25^ *Rac2* encodes a member of the *Ras* superfamily of guanosine triphosphate (GTP) metabolic proteins that regulate a variety of processes, including secretion, cell polarization, and phagocytosis.^26^ In the present study, *Rac2* was identified as significantly enriched in several pathways, including natural killer cell‐mediated cytotoxicity.^27^ Furthermore, *Tyrobp* and *Rac2* have been found to be key genes that regulate immune and inflammatory responses after SCI.^28^ *Ras* GTPases play a key role in multiple processes in which SCI occurs. Inhibition of neural stem cells is a promising treatment for SCI, and the study of Numano et al. demonstrated that inhibition of *Rac2* expression can improve the survival rate of transplanted stem cells.^29^ *Cd68* encodes a 110‐kD transmembrane glycoprotein that is highly expressed by human monocytes and tissue macrophages.^30^ The protein is also a member of the scavenger receptor family, typically function to clear cellular debris, promote phagocytosis and mediate the recruitment and activation of macrophages.^31^ The *Aif1* gene is induced by cytokines and interferon (INF) and may promote macrophage activation and growth of vascular smooth muscle cells and T‐lymphocytes.^32^ The protein encoded by this gene associates with the class II MHC and is an important chaperone that regulates antigen presentation for immune response. It also serves as a cell surface receptor for the cytokine macrophage migration inhibitory factor (MIF), which, when bound to the encoded protein, initiates survival pathways and cell proliferation.^33^ The *C1qb* gene encodes the B‐chain polypeptide of serum complement subcomponent C1q, which associates with C1r and C1s to yield the first component of the serum complement system. Byrnes et al. compared gene clusters at two different time points after SCI in rats and found that reduced expression of *C1qb* was associated with microglial activation, regulating secondary injury and recovery of SCI from cytotoxicity and neuroprotection.^34^ The *Spi1* gene encodes an ETS‐domain transcription factor that activates gene expression during myeloid and B‐lymphoid cell development.^35^ *Fcer1g*, a high‐affinity IgE receptor, is a key molecule involved in allergic reactions.^36^ The proteins encoded by *RT1‐DA* and *Ccl4* were also associated with inflammatory function.^37^, ^38^ Also, immune response and inflammatory response are the key pathological processes after SCI.^28^ Jogia retrospectively analyzed 161 patients with SCI and found that early control of SCI‐induced systemic immune response was beneficial to longer‐term recovery.^39^ Therefore, combined with the results, the promoting effect of the WTM test on the recovery of motor function in rats with SCI is most likely to be achieved by reducing the expression of the above genes and thus reducing the inflammatory response of rats after SCI.

### 4.3 GO enrichment and KEGG pathways

The highest concentration score terms of BP, CC, and MF were positive regulation of microglial cell‐mediated cytotoxicity and MHC, particularly the MHC class II protein complex. GO enrichment and KEGG pathway enrichment analyses of 133 proteins showed that the proteins were mainly enriched in microglia‐mediated cytotoxicity, MHC class II protein complex and MHC class II protein complex binding. This suggested that the WTM test may promote the recovery of motor function in rats by regulating the cell–cell communication and immune function.

Nuclear factor‐κB (NF‐κB) is considered a central transcription factor of inflammatory mediators and plays a key role in SCI, leading to microglial activation, inflammatory response, and ultimately neuronal death, resulting in permanent neurological impairment.^40^ Neuroinflammation caused by microglia is an important contributing factor to the formation of secondary injury induced by SCI.^41^ SCI induces the release of inflammatory factors such as interleukin (IL), TNF, and INF, which directly lead to neuronal death while inducing vascular endothelial cells to express various cell adhesion and chemotactic molecules. These proinflammatory factors stimulate nitric oxide synthesis, lead to increased capillary permeability and blood–spinal barrier dysfunction and promote neuronal apoptosis. Inhibition of microglia activation and subsequent neuroinflammatory response have been shown to promote recovery in patients with SCI.^41^ The WTM test may regulate microglia‐induced inflammation by regulating the expression of *Tyrobp, Rac2*, and other genes.^42^

MHC is a group of closely linked genes located on the short arm of human chromosome 6, encoding the major histocompatibility antigen, which is involved in the immune response and immune regulation of the body, and determines tissue compatibility. MHC class II molecules are mainly expressed on the surface of antigen‐presenting cells such as B cells, mononuclear macrophages, and dendritic cells, and their main function is to present the processed antigen fragments to CD4+ T cells during the immune response to generate immune response.^43^ Microglia is a kind of phagocytic macrophage with abundant expression of MHC class II molecules on its surface, and plays an important role in maintaining the stability of the CNS and monitoring, maintenance, and protection of its functions.^44^ When stimulated, microglia can be rapidly activated and releases a variety of inflammatory mediators, such as cytokines, chemokines, nitric oxide, and reactive oxygen species. All of these results suggested that the WTM test may promote the recovery of motor function in rats by regulating these proteins and related signaling pathways, and then regulating intercellular communication and immune function.

## 5 CONCLUSION

The results of BBB scores showed that WTM could promote the recovery of SCI motor function. WTM may further reduce inflammation and promote the recovery of nerves and blood vessels by regulating the expression of genes such as *Tyrobp, Rac2, Cd68, C1qb, Aif1, Cd74, Spi1, Fcer1g, RT1‐DA*, and *Ccl4*, thus promoting the recovery of motor function in rats with SCI. All of these genes are involved in immunity and biological regulation, such as *Rac*2, which is enriched in T natural killer cell‐mediated cytotoxicity. The results of KEGG and GO indicate that the therapeutic effects of WTM on motor function in rats may be mediated by the positive regulation of microglia‐mediated cytotoxicity. MHC class II protein complex binding and NF‐κB regulate intercellular communication and immune function in rats with SCI. These results indicate that the WTM test plays an important role in SCI recovery, but the relevant mechanism remains to be further confirmed by research.

## CONFLICT OF INTERESTS

The authors declare that there are no conflict of interests.

## ETHICS STATEMENT

All experimental procedures were approved by the Animal Care & Welfare Committee of Kunming Medical University (kmmu2019005).

## AUTHOR CONTRIBUTIONS

**Bao‐Lei Zhang**: conceptualization, methodology. **Qiu‐Lin Wang**, **Chang‐Le Fang**: data curation, writing original draft preparation; **Ting‐Ting Li**: complete the figures and revised draft.

## Data Availability

The data used to support the findings of this study are available from the corresponding author upon request.
